# Antiviral therapy inhibited HBV-reactivation and improved long-term outcomes in patients who underwent radiofrequency ablation for HBV-related hepatocellular carcinoma

**DOI:** 10.1186/s12957-023-02921-1

**Published:** 2023-02-11

**Authors:** Jian Liu, Hao Shen, Shengyu Huang, Jianbo Lin, Zhenlin Yan, Guojun Qian, Zhenghua Lu, Xuying Wan, Fabiao Zhang, Kui Wang, Yongjie Zhang, Jun Li

**Affiliations:** 1 Department of Hepatic Surgery, The Eastern Hepatobiliary Surgery Hospital, Naval Medical University, Shanghai, China; 2 Department of Biliary Surgery, The Eastern Hepatobiliary Surgery Hospital, Naval Medical University, Shanghai, China; 3 Department of Hepatobiliary and Pancreatic Surgery, Tenth People’s Hospital of Tongji University, Shanghai, China; 4 Department of Minimally Intervention Therapy, The Eastern Hepatobiliary Surgery Hospital, Naval Medical University, Shanghai, China; 5 Department of Clinical Database, The Eastern Hepatobiliary Surgery Hospital, Naval Medical University, Shanghai, China; 6 Taizhou Hospital of Zhejiang Province, Affiliated to Wenzhou Medical University, Taizhou, China

**Keywords:** Hepatocellular carcinoma, Radiofrequency ablation, Antiviral therapy, HBV reactivation, Prognosis

## Abstract

**Background:**

Hepatitis B virus (HBV) reactivation impact negatively the prognosis of patients with HBV-related hepatocellular carcinoma (HCC). This study aimed to observe the effect of antiviral therapy (AVT) on viral reactivation and long-term outcomes after percutaneous radiofrequency ablation (PRFA) for HBV-related HCC.

**Methods:**

Data on 538 patients between 2009 and 2013 were reviewed. Propensity score matching (PSM) analysis was used to adjust for differences in baseline features between patients who received AVT (AVT group) and did not receive it (non-AVT group). Logistic regression was used to identify the independent factors for viral reactivation. The tumor recurrence and overall survival (OS) rates were analyzed using the Kaplan–Meier method. Recurrence patterns were also investigated.

**Results:**

HBV reactivation developed in 10.8% (58/538) of patients after PRFA. AVT was associated independently with decreased viral reactivation (odd ratio: 0.061, 95% confidence interval: 0.018–0.200). In 215 pairs of patients obtained after PSM, the AVT group had lower 1-, 3-, and 5-year recurrence rates (24%, 55%, and 67% vs 33%, 75%, and 85%, respectively) and higher 1-, 3-, and 5-year OS rates (100%, 67%, and 59% vs 100%, 52%, and 42%, respectively) than non-AVT group (*P* < 0.001 for both). Additionally, the relapses in distant hepatic segments and the late recurrence after 2 years of PRFA were significantly reduced in the AVT group (78/215 vs 111/215 vs., *P* = 0.001; 39/109 vs. 61/91, *P* = 0.012, respectively).

**Conclusions:**

AVT reduced late and distal intrahepatic recurrence and improved OS in patients undergoing PRFA for HBV-related HCC by inhibiting viral reactivation.

**Supplementary Information:**

The online version contains supplementary material available at 10.1186/s12957-023-02921-1.

## Introduction

Hepatocellular carcinoma (HCC) is the fifth most common malignancy and the second leading cause of cancer-related death worldwide [1]. Hepatitis B virus (HBV) infection is the major causative factor of HCC in high-prevalence regions [2, 3]. Percutaneous radiofrequency ablation (PRFA) is one of the first-line treatment options, in addition to liver transplantation and liver resection, for early-stage HCC, especially for older individuals and patients with underlying diseases not suitable for surgery [4]. Unfortunately, the tumor recurrence is still common, developing in about 70% of patients within 5 years post-PRFA [5]. Compared with liver resection, PRFA has been reported to have an increased risk of local tumor recurrence [4].

Viral reactivation may occur in patients who suffer from both HBV infection and HCC or other malignancies when they undergo systemic chemotherapy, radiotherapy, surgical resection, and organ transplantation, resulting in a sustained liver damage and possibly a poorer long-term prognosis [6–10]. Antiviral therapy (AVT) has been reported to suppress viral reactivation and improve long-term prognoses [7–12]. Anti-recurrence role of AVT has also been documented in patients who received PRFA for HBV-related HCC [13, 14]. However, there were few studies regarding the effect of AVT on viral reactivation after PRFA. In addition, the impact of AVT on the recurrence patterns, including intrahepatic distribution of recurrent nodules and time interval between primary and recurrent tumors, was not investigated, which was also an important issue to illustrate the role of HBV in tumorigenesis [14].

Therefore, the current study aimed to examine the impact of AVT on short- and long-term outcomes, including viral reactivation, recurrence rate, recurrence patterns, and overall survival, in patients undergoing PRFA for HBV-related HCC. We present this study in accordance with the STROBE reporting checklist.

## Methods

### Study population

**Fig. 1:**
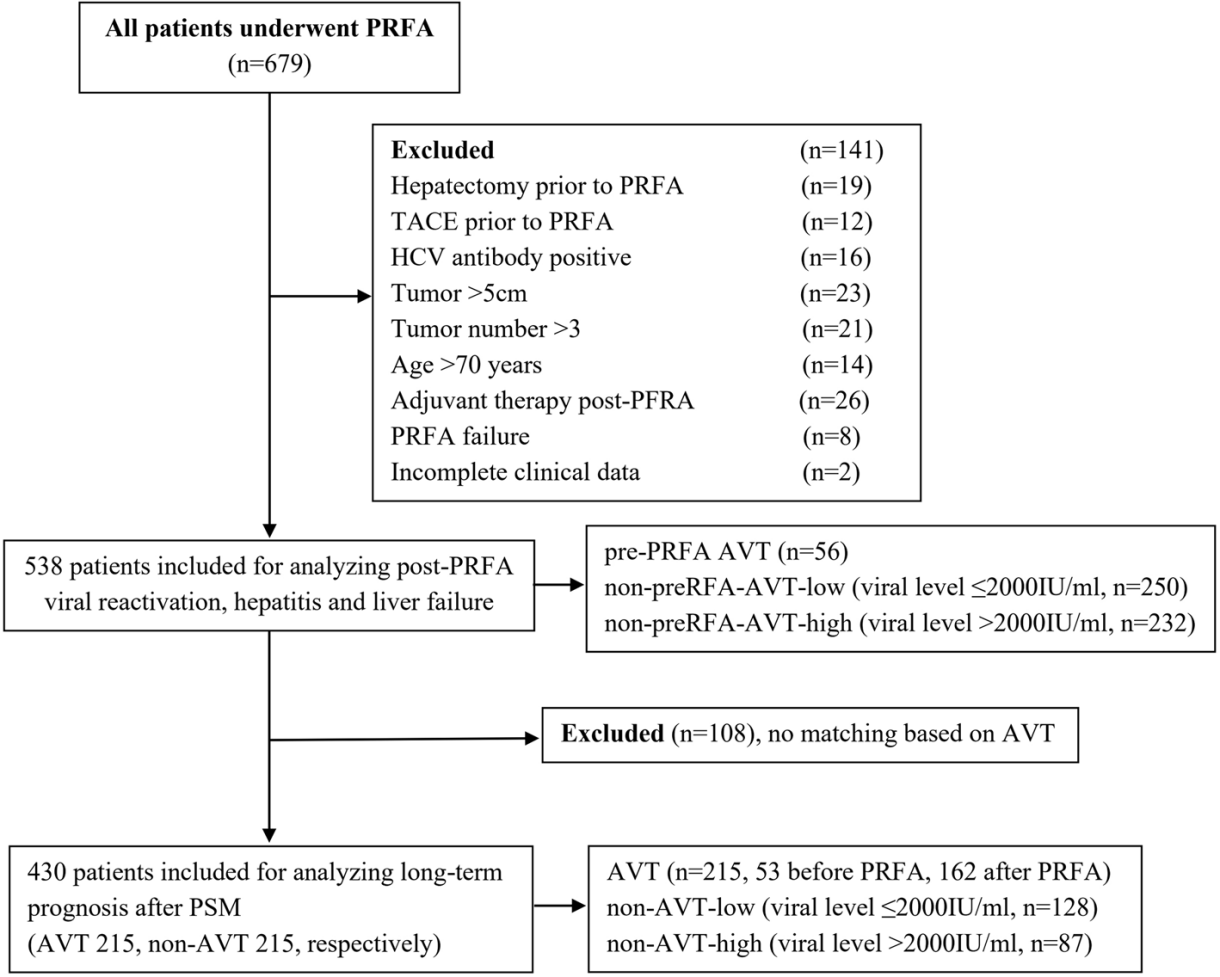
Study flow diagram

Data on 679 consecutive patients who underwent PRFA as the first-line treatment for HCC between February 2009 and October 2013 at the Eastern Hepatobiliary Surgery Hospital (EHBH) were prospectively collected and retrospectively reviewed. Patients were included if they met the following criteria: (1) age between 20 and 70 years; (2) hepatitis B surface antigen (HBsAg) and/or hepatitis B core antibody (HBcAb) positivity; (3) Barcelona Clinic Liver Cancer (BCLC) 0-A stage [15]; (4) complete ablation demonstrated by imaging studies (absence of enhancing area) and serology findings (negative level after ablation in patients with a positive level of alpha-fetoprotein [AFP] before treatment) [5]; (5) did not receive anti-cancer treatment prior to PRFA; (6) did not receive adjuvant therapy including transarterial chemoembolization (TACE), radiotherapy, or chemotherapy after PRFA; and (7) without a history of other malignancies. Patients who had concomitant hepatitis C virus (HCV) infection and/or received additional treatment due to ablative failure and/or had incomplete clinical data were excluded [16]. Accordingly, as shown in Fig. 1, a total of 141 patients were excluded the remaining 538 patients were further analyzed. This study was approved by the Institutional Ethics Committee of the EHBH, and the written consent was obtained from all patients for using their data in the research.

### Pre-treatment work-up and PRFA

Before PRFA, patients underwent routine serological examinations including liver function tests, hepatitis B and C antigens/antibodies, HBV deoxyribonucleic acid (HBV DNA), and AFP. The imaging studies included chest radiography, abdominal ultrasound, and contrast-enhanced computerized tomography (CT) scan and/or magnetic resonance imaging (MRI) of the abdomen. The clinical diagnosis of HCC was according to the criteria proposed by the American Association for the Study of Liver Diseases [15].

The therapeutic decision was made through discussions within a multidisciplinary team which usually included hepatic surgeons, interventional radiologists, and hepatologists. PRFA was indicated following the previously proposed criteria [17]. Briefly, the indications were (1) Eastern Cooperative Oncology Group (ECOG) Performance score of 0 to 2; (2) tumor ≤ 5 cm in size, ≤ 3 nodules; (3) no any pieces of evidence of extrahepatic distant metastasis; (4) no major portal/hepatic veins invasion; (5) nodules located beyond 0.5 cm far from the gallbladder, colon, stomach, or common bile duct based on imaging study findings; (6) Child–Pugh grade A or B; (7) no severe coagulopathy (prolongation of prothrombin time > 5 s) or no severe thrombocytopenia (platelet count < 40 × 10^9^/L); and (8) without ascites or with ascites which was well controlled before treatment.

The PRFA was carried out as previously reported [16]. It was performed under the guidance of ultrasound by operators who had more than 10 years of operating experience. A Cool-tip RF ablation system (Valleylab, Boulder, CO) was used to achieve a single ablation or multiple overlapping ablations based on tumor size, aiming to obtain a transient hyper-echoic zone covering an area larger than the entire lesion. A dynamic CT scan was performed 4 weeks after each treatment session to evaluate the efficacy of ablation [17]. On CT images, any non-enhancing area was considered as the ablated zone. When the non-enhancing area was larger than the primary nodule, PRFA was complete. In contrast, when the non-enhancing area was similar to the primary nodule without any margin or when partial enhancement of any part of the ablated tumor was observed, PRFA was considered to be incomplete. An additional session of ablation was accordingly performed. Residual viable tumor lesions were identified at 1 month after ablation if enhancement areas on the CT scan were seen within the tumor. MRI was carried out if CT was uncertain about whether there was a residual viable tumor lesion. Additional treatment with RFA or percutaneous ethanol injection was given for residual tumors. If a residual viable tumor was still present after repeated treatments, the PRFA was deemed to be a failure and further salvage hepatectomy or liver transplantation or TACE was performed. In this study, 2, 2, and 3 patients experienced TACE, transplantation, and resection due to the failure of PRFA, respectively [16].

### Antiviral treatment

Antiviral treatment was defined as reported [3]. All antiviral medication used in this study was nucleotide/nucleoside analogs, which included lamivudine (100 mg per day, GlaxoSmithKline), adefovir (10 mg per day, GlaxoSmithKline), and entecavir (0.5 mg per day, Sino-American Squibb). Patients who received effective AVT before PRFA were usually recommended with the same medication after PRFA.

### Definitions

As previously reported, viral reactivation was defined by either an increase of more than 10 folds in serum viral level when compared with the baseline level for patients with detectable viral load or serum viral level of more than 200 IU/mL for patients with undetectable viral level within three months after PRFA [11]. The viral level was tested repeatedly when viral reactivation occurred.

Post-PRFA hepatitis was defined as a significant and sustained abnormality in serum alanine transaminase (ALT) level after treatment. The upper limit of normal (ULN) of ALT was ≤ 40 IU/L. The increase in ALT by < 3 × ULN was defined as mild, 3 to 5 × ULN as moderate, and > 5 × ULN as severe hepatitis [11].

The different types of intrahepatic recurrence were defined as follows: local recurrence in the ablation zone is defined as any recurrence within 1 cm of the ablation zone after PRFA, irrespective of additional re-recurrence in other parts of the liver; recurrence in the adjacent segment is defined as any recurrence in the adjacent segment or in the same segment of the primary tumor and beyond 1 cm away from ablation zone; recurrence in distant segment refers to any recurrence that is not in the adjacent segment or in the contralateral hemiliver; recurrences in multi-segments indicate multiple recurrences involving more than two hepatic segments [17]. Early and late recurrence were defined as recurrence diagnosed within or beyond 2 years of PRFA, respectively.

The definition of AVT required the patients to receive at least one kind of NAs for three consecutive months, that was similar to previous studies on HBV-related patients with HCC [12].

### Follow-up and endpoints

Patients were followed up every 1–2 months during the first 2 years and every 3–6 months thereafter, using the protocol and methods as previously reported [18]. Liver function test, AFP level, HBV DNA, and abdominal ultrasound were checked at each of the visits. AVT was advised if viral reactivation was observed for patients who did not use it previously. For all AVT patients, they were informed clearly of the necessity of long-term treatment and that the discontinuation of treatment for any reason should consult a doctor. On each follow-up visit, the efficacy of AVT was verified to assess drug resistance [12].

The follow-up was ended in December 2021. The endpoints of the study included tumor recurrence and overall survival (OS). Tumor recurrence was calculated from the date of PRFA to the date when the first recurrence/metastasis was diagnosed. OS was measured from the date of PRFA to the date of death or the last follow-up. The post-PRFA viral reactivation and the patterns of tumor recurrence were also observed.

### Statistical analyses

The *χ*^2^ test or Fisher’s exact test was used to compare qualitative variables, while continuous variables were compared using Student’s *t* test or Mann–Whitney test for variables with an abnormal distribution. Logistic regression analysis was used to determine the factors of viral reactivation. Survival curves were calculated using the Kaplan–Meier method and compared by log-rank test. The Cox proportional hazards model was used to determine the independent factors for tumor recurrence and OS.

The propensity score matching (PSM) analysis was carried out to reduce the differences in baseline data between patients who received AVT (AVT group) and those who did not receive it (non-AVT group). By using multiple logistic regression analysis, a propensity score was estimated for all patients treated with AVT. Caliper matching was performed on the PSM (nearest available matching). Pairs of AVT and non-AVT patients on the PSM logit were matched to within a range of 0.2 standard deviations. The sample size needed for further analysis after 1:1 PSM was calculated according to the difference in the 5-year recurrence rate between the AVT group and the non-AVT group. As reported, the 5-year recurrence rate of the non-AVT group was about 80% and we estimated that it would decrease by 15% (from 80 to 65%) in the AVT group. The *α* error was set at 0.05, and the power was 0.90. Finally, the planned sample size was 396 (198 patients in each group were required) based on a two-sided Fisher’s exact test.

Data analysis was performed using IBM SPSS software (Armonk, NY, version 19.0) and R software (R Foundation for Statistical Computing, Vienna, Austria; www.r-project.org, version 3.4.1). A two-sided *P* value < 0.05 was considered statistically significant.

## Results

### Patient characteristics

In the entire cohort of 538 patients, 240 (240/538, 44.6%) patients received AVT, with 56 (56/538, 10.4%) initiated before PRFA, 156 (156/538, 29.0%) immediately after PRFA, and 28 (28/538, 5.2%) after the presence of viral reactivation. Detailed information on AVT medication is listed in Supplementary Table 1. The proportions of patients with positive HBsAg and hepatitis B e antigen (HBeAg) between the AVT and non-AVT groups were different (Supplementary Table 2). After 1:1 PSM using variables of HBsAg, HBeAg, and alkaline phosphatase (Supplementary Table 3), a new cohort of 215 pairs of patients was created in the AVT and non-AVT groups. The baseline features were balanced between the two groups (Table 1).

**Table 1:** Baseline characteristics in cohort generated by PSM

Variables	Median (IQR)/number		*P*
	**AVT (*n* = 215)**	**Non-AVT (*n* = 215)**	
Age, years	55 (29–70)	54 (28–70)	0.663
Sex, female: male	30: 185	29: 186	0.889
Diabetes mellitus, yes: no	33: 182	24: 191	0.201
AFP, μg/L ≥ 20, yes: no	95: 120	104: 111	0.384
Tumor number, single: multiple	162: 53	163: 52	0.911
Liver cirrhosis, yes: no	51: 164	52: 163	0.910
Diameter, cm	2.4 (0.8–5.0)	2.4 (0.9–5.0)	0.221
BCLC staging 0: 1	59: 156	52: 163	0.440
ECOG, 0: 1: 2	102: 107: 6	93: 116: 6	0.678
Total bilirubin, μmol/L	14.6 (3.5–50.4)	15.1 (6.3–49.4)	0.437
Albumin, g/L	41.5 (27.1–52.8)	42 (26.1–52.1)	0.626
Platelets, 10^9^/L	127 (40–372)	118 (41–295)	0.092
Prothrombin time, s	12.5 (10.1–16)	12.4 (10.3–15.6)	0.829
GGT, U/L	48 (9–762)	57 (7–844)	0.069
ALP, U/L	80 (16–273)	83 (29–443)	0.357
ALT, U/L	31.4 (7.9–212.3)	31.3 (6.2–290.1)	0.538
Creatinine, μmol/L	67 (4–155)	68 (28–273)	0.517
AFU, U/L	25 (6–57)	26 (5–60)	0.635
HBsAg, positive: negative	210: 5	210:5	1.000
HBeAg, positive: negative	55: 160	55: 160	1.000
HBV-DNA, IU/mL, ≥ 2000: < 2000	85: 130	87: 128	0.844
HBV reactivation, yes: no	27: 188	22: 193	0.448

*Abbreviations: IQR* Interquartile range, *AVT* Antiviral therapy, *AFP* Alpha-fetoprotein, *BCLC* Barcelona Clinic Liver Cancer, *ECOG* Eastern Cooperative Oncology Group score standard, *GGT* Gamma-glutamyl transferase, *ALP* Alkaline phosphatase, *ALT* Alanine aminotransferase, *AFU* α-L-fucosidase, *HbsAg* Hepatitis B surface antigen, *HBeAg* Hepatitis B e antigen

### Viral reactivation and long-term prognosis in the entire cohort

In the entire cohort, 58 (10.8%) patients experienced viral reactivation while 480 patients did not. Patients with HBV-DNA level ≥ 2000 IU/mL had an increased incidence of viral reactivation when compared with patients who had HBV-DNA level of < 2000 IU/mL (16.4 vs. 6.5%). The multivariable logistic analysis identified that HBeAg positivity (odds ratio [OR] 2.403, 95% confidence interval [CI], 1.291–4.474), HBV-DNA level ≥ 2000 IU/mL (2.233, 1.213–4.111) and AVT (0.061, 0.018–0.200) were independently associated with viral reactivation after PRFA (Table 2).

**Table 2:** Analysis of independent risk factors for viral reactivation

Variable	Univariate			Multivariate		
	**OR**	**95% CI**	***P***	**OR**	**95% CI**	***P***
Age, years	1.151	0.666–1.991	0.614			
Sex, female: male	1.554	0.784–3.081	0.207			
Diabetes mellitus, yes: no	0.737	0.304–1.784	0.498			
AFP, μg/L ≥ 20, yes: no	1.655	0.955–2868	0.073			
Tumor number, single: multiple	1.237	0.670–2.284	0.496			
Liver cirrhosis, yes: no	1.045	0.553–1.978	0.891			
Diameter, cm, ≤ 3: > 3	0.672	0.320–1.414	0.295			
BCLC staging 0: 1	1.206	0.629–2.310	0.573			
ECOG, 0: 1: 2	1.503	0.911–2.480	0.111			
Total bilirubin, μmol/L	1.647	0.928–2.923	0.088			
Albumin, g/L	0.634	0.304–1.324	0.225			
Platelets, 10^9^/L	0.850	0.486–1.485	0.568			
Prothrombin time, s	1.051	0.588–1.880	0.866			
GGT, U/L	0.720	0.413–1.256	0.247			
ALP, U/L	0.753	0.329–1.723	0.502			
ALT, U/L	0.837	0.461–1.520	0.559			
Creatinine, μmol/L	0.994	0.979–1.009	0.445			
AFU, U/L	0.764	0.226–2.5774	0.664			
HBsAg, positive: negative	3.039	0.719–12.856	0.131			
HBeAg, positive: negative	2.319	1.320–4.076	0.003	2.403	1.291–4.474	0.006
HBV-DNA, IU/mL, ≥ 2000: < 2000	2.801	1.582–4.959	< 0.001	2.233	1.213–4.111	0.010
AVT before reactivation, yes: no	0.071	0.022–0.229	< 0.001	0.061	0.018–0.200	< 0.001

*Abbreviations: QR* Odds ratio, *CI* Confidence interval, *AFP* Alpha-fetoprotein, *BCLC* Barcelona Clinic Liver Cancer, *ECOG* Eastern Cooperative Oncology Group score standard, *GGT* Gamma-glutamyl transferase, *ALP* Alkaline phosphatase, *ALT* Alanine aminotransferase, *AFU* α-L-fucosidase, *HbsAg* Hepatitis B surface antigen, *HBeAg* hepatitis B e antigen, *AVT* Antiviral therapy

**Fig. 2:**
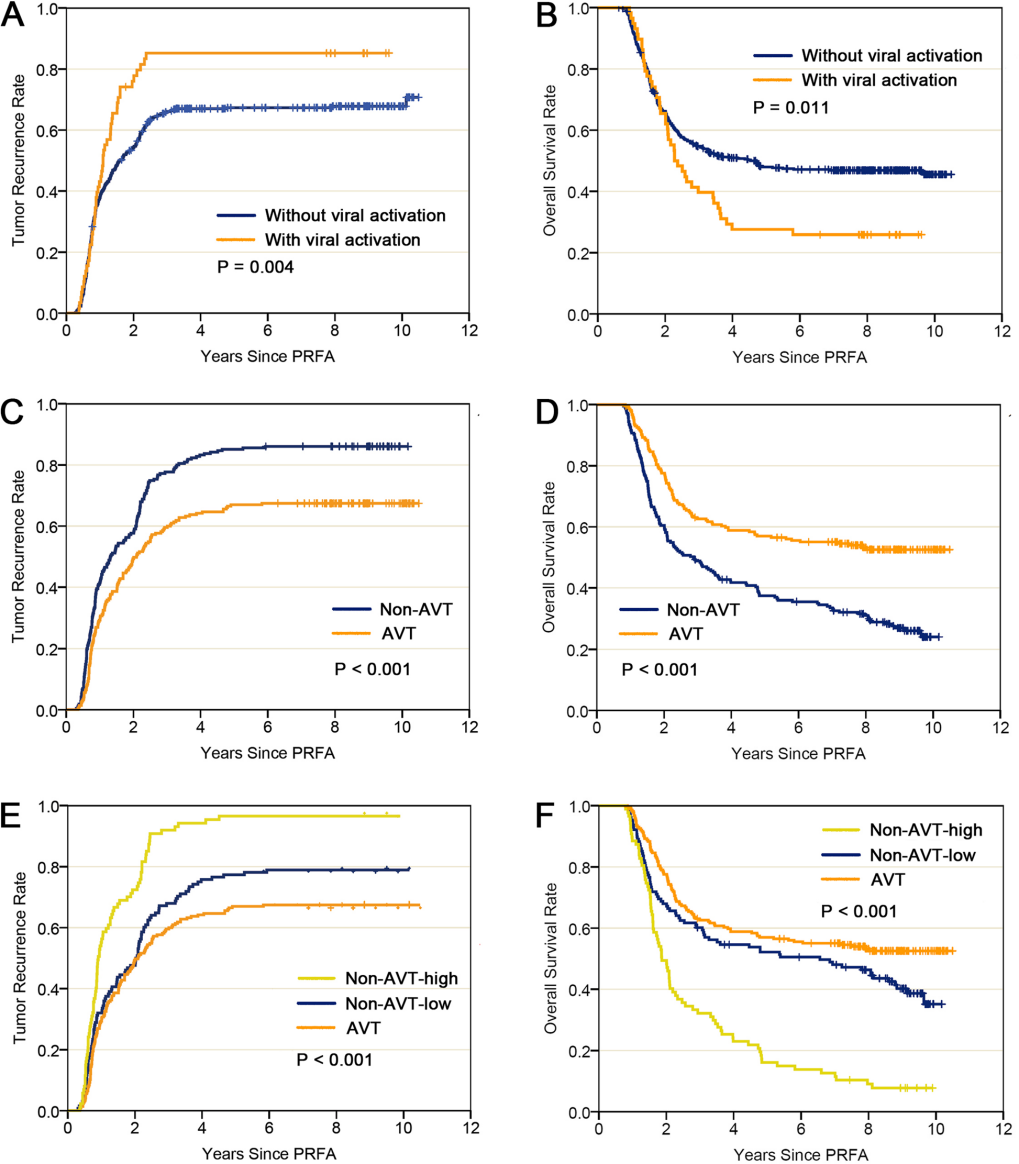
**A**, **B** Tumor recurrence and overall survival between patients with viral reactivation and without viral reactivation after PRFA for HBV-related hepatocellular carcinoma in the entire cohort. **C**, **D** Tumor recurrence and overall survival between patients receiving antiviral treatment (AVT) and not receiving AVT (non-AVT) after PRFA for HBV-related hepatocellular carcinoma. **E**,** F** Tumor recurrence and overall survival among three groups of patients receiving antiviral treatment (AVT), not receiving AVT with a low viral level (non-AVT-low) and not receiving AVT with a high viral level (non-AVT-high) after PRFA for HBV-related hepatocellular carcinoma (tumor recurrence: AVT vs. non-AVT-high, *P* < 0.001; AVT vs. non-AVT-low, *P* < 0.001; non-AVT-high vs. non-AVT-low, *P* = 0.037; overall survival: AVT vs. non-AVT-high, *P* < 0.001; AVT vs. non-AVT-low, *P* < 0.001; non-AVT-high vs. non-AVT-low, *P* = 0.046)

The median follow-up duration in the entire cohort was 45.9 months (range: 8.9–125.9 months). The 1-, 3-, and 5-year recurrence rates for patients with viral reactivation were significantly higher than those without reactivation (47%, 85%, and 85% vs. 41%, 66%, and 70%, *P* = 0.004) (Fig. 2A). The corresponding OS rates were significantly lower in patients with viral reactivation (97%, 36%, and 21% vs. 94%, 53%, and 42%, *P* = 0.011) (Fig. 2B).

### Survival analysis in the PSM-matched cohort

In the PSM-matched cohort, 330 recurrences were identified with 1-, 3-, and 5-year recurrence rates of 29%, 65%, and 74%, respectively, and 256 deaths occurred with 1-, 3-, and 5-year survival rates of 100%, 59%, and 50%, respectively. In the non-AVT group, the 1-, 3-, and 5-year recurrence rates were significantly higher than those in the AVT group (33%, 75%, and 85% vs. 24%, 55%, and 67%, respectively, *P* < 0.001, Fig. 2C). Meanwhile, the OS rates in the non-AVT group was significantly lower than those AVT group (100%, 52%, and 42% vs. 100%, 67%, and 59%, respectively, *P* < 0.001, Fig. 2D).

The PSM cohort was also stratified into three groups: the non-AVT-low group (viral level < 2000 IU/mL, *n* = 128), non-AVT-high group (≥ 2000 IU/mL, *n* = 87), and AVT group (*n* = 215). The 1-, 3-, and 5-year recurrence rates for the three groups were 32.0%, 68.0%, and 77.3%; 55.2%, 92.0%, and 96.6%; 29.3%, 59.5%, and 67.0%, respectively, and the corresponding OS rates were 95.3%, 60.2%, and 52.3%; 88.5%, 32.2%, and 16.1%; 98.1%, 62.8%, and 57.2%, respectively (*P* < 0.001 for both, Fig. 2E, F). The AVT group had the highest OS rates and lowest recurrence rates compared with the other two groups.

As shown in Table 3, the independent factors associated with tumor recurrence included tumor size ≥ 3 cm (hazard ratio [HR] 1.155, 95% CI, 1.013–1.316), multiple tumors (1.524, 1.187–1.957), cirrhosis (1.710, 1.344–2.175), HBV-DNA level ≥ 2000 IU/mL (1.377, 1.097–1.729), viral reactivation (1.427, 1.014–2.009), HBsAg positivity (5.096, 1.256–20.667), and AVT (0.578, 0.463–0.722). In the analysis for OS, the independent factors were cirrhosis (HR, 1.744, 95%CI, 1.334–2.280), viral level ≥ 2000 IU/mL (1.566, 1.208–2.029), and AVT (0.509, 0.395–0.657).

**Table 3:** Analysis of independent risk factors for tumor recurrence and overall survival

Variables	Tumor recurrence						Overall survival					
	**Univariate**			**Multivariate**			**Univariate**			**Multivariate**		
	**HR**	**95% CI**	***P***	**HR**	**95% CI**	***P***	**HR**	**95% CI**	***P***	**HR**	**95% CI**	***P***
Age, years	1.002	0.991–1.013	0.759				0.999	0.987–1.012	0.901			
Sex, female: male	1.098	0.811–1.487	0.546				0.947	0.657–1.365	0.771			
Diabetes mellitus, yes: no	1.171	0.862–1.590	0.313				1.064	0.745–1.520	0.732			
AFP, μg/L ≥ 20, yes: no	1.148	0.925–1.425	0.212				1.174	0.919–1.501	0.200			
Tumor number, single: multiple	1.556	1.220–1.986	< 0.001	1.524	1.187–1.957	0.001	1.340	1.017–1.767	0.038	1.220	0.922–1.614	0.163
Liver cirrhosis, yes: no	1.821	1.436–2.308	< 0.001	1.710	1.344–2.175	< 0.001	1.887	1.450–2.456	< 0.001	1.744	1.334–2.280	< 0.001
Diameter, cm, ≤ 3: > 3	1.142	1.009–1.293	0.036	1.155	1. 013–1.316	0.031	1.076	0.939–1.234	0.290			
BCLC staging 0: 1	1.079	0.842–1.382	0.547				0.977	0.740–1.289	0.867			
ECOG, 0: 1: 2	1.042	0.856–1.269	0.682				0.997	0.798–1.245	0.978			
Total bilirubin, μmol/L	1.011	0.998–1.025	0.100				1.015	1.001–1.030	0.040	1.002	0.985–1.020	0.794
Albumin, g/L	0.978	0.955–1.001	0.064				0.979	0.954–1.005	0.110			
Platelets, 109/L	0.998	0.996–1.000	0.034	0.877	0.699–1.099	0.255	0.997	0.995–0.999	0.006	0.999	0.996–1.001	0.999
Prothrombin time, s	1.120	0.970–1.532	0.090				1.130	1.015–1.258	0.025	1.033	0.904–1.180	0.637
GGT, U/L	1.001	1.000–1.002	0.068				1.001	1.000–1.002	0.099			
ALP, U/L	1.002	0.999–1.004	0.143				1.001	0.999–1.004	0.269			
ALT, U/L	0.999	0.996–1.002	0.646				0.998	0.994–1.002	0.281			
Creatinine, μmol/L	0.997	0.991–1.003	0.378				1.001	0.995–1.007	0.740			
AFU, U/L	1.011	0.999–1.023	0.086				1.019	0.905–1.032	0.066			
HBsAg, positive: negative	6.404	1.594–25.732	0.009	5.096	1.256–20.667	0.023	4.051	1.007–16.291	0.049	2.477	0.608–10.101	0.206
HBeAg, positive: negative	1.009	0.788–1.293	0.941				0.940	0.707–1.249	0.668			
HBV-DNA, IU/mL, ≥ 2000: < 2000	1.528	1.229–1.901	< 0.001	1.377	1.097–1.729	0.006	1.660	1.298–2.123	< 0.001	1.566	1.208–2.029	0.001
AVT, yes: no	0.607	0.488–0.755	< 0.001	0.578	0.463–0.722	< 0.001	0.519	0.404–0.667	< 0.001	0.509	0.395–0.657	< 0.001
HBV reactivation, yes: no	1.475	1.062–2.049	0.020	1.427	1.014–2.009	0.041	1.477	1.041–2.094	0.029	1.377	0.954–1.987	0.088

*Abbreviations: HR* Hazard ratio, *CI* Confidence interval, *AFP* Alpha-fetoprotein, *BCLC* Barcelona Clinic Liver Cancer, *ECOG* Eastern Cooperative Oncology Group score standard, *GGT* Gamma-glutamyl transferase, *ALP* Alkaline phosphatase, *ALT* Alanine aminotransferase, *AFU* α-L-fucosidase, *HbsAg* Hepatitis B surface antigen, *HBeAg* hepatitis B e antigen, *AVT* Antiviral therapy

### Impact of AVT on patterns of recurrence

As shown in Table 4, there was no significant difference in the types of recurrence (intrahepatic, extrahepatic, and intrahepatic plus extrahepatic) observed between the AVT and non-AVT groups (*P* = 0.992). In the further analysis of the site of intrahepatic recurrent nodules, recurrence in the distant hepatic segment was more commonly seen in the non-AVT group than AVT group (111/215 vs. 78/215, *P* = 0.001), but there was no significant difference in local recurrence (13/215 vs. 8/215, *P* = 0.263), recurrence in the adjacent segment (43/215 vs. 37 /215, *P* = 0.457) or in multi-segments (9/215 vs. 6/215, *P* = 0.430).

**Table 4:** Patterns of tumor recurrence

Parameter	AVT group (*n* = 215)	Non-AVT group (*n* = 215)	*P*
**Type of recurrence**	145	185	0.992
Intrahepatic^a^ (*n* = 305)	134	171	
Extrahepatic^b^ (*n* = 11)	5	6	
Intrahepatic plus extrahepatic^c^ (*n* = 14)	6	8	
**Site of intrahepatic recurrence**^d^	134	171	0.312
Local (*n* = 21)	13	8	0.263
Adjacent segment (*n* = 80)	37	43	0.457
Distant segment (*n* = 189)	78	111	0.001
Multi-segments (*n* = 15)	6	9	0.430
**Time to recurrence, months**	145	185	
≤ 24 (*n* = 230)	106	124	0.082
> 24 (*n* = 100)	39	61	0.012

^a^Included intrahepatic recurrence only

^b^Extrahepatic recurrence only

^c^Intra-plus extrahepatic recurrence

^d^Local recurrence is defined as any recurrence at the ablation zone after PRFA within 1 cm, irrespective of additional recurrence in other parts of the liver; recurrence in the adjacent segment is defined as any recurrence in the adjacent segment or in the same segment 1 cm away from ablation zone; recurrence in distant segment refers to any recurrence that was not in the adjacent segment or in the contralateral hemiliver; recurrence in multi-segments indicates multiple recurrences involving more than two hepatic segments

The independent factors associated with local recurrence **(**21/430, 4.9%) and recurrence in the distant segment (284/430, 66.0%) were further analyzed. The tumor size > 3 cm was the only factor associated with an increased risk of local recurrence (HR 2.664, 95% CI, 1.425–4.980, *P* = 0.002) (Supplementary Table 4), while multiple tumors (1.467, 1.129–1.906, *P* = 0.004), cirrhosis (1.757, 1.362–2.267, *P* < 0.001), HBV-DNA ≥ 2000 IU/mL (1.408, 1.107–1.791, *P* = 0.006), viral reactivation (1.548, 1.087–2.204, *P* = 0.015), and AVT (0.569, 0.450–0.719, *P* < 0.001) were associated with recurrence in the distant segment (Supplementary Table 5).

Additionally, there was no significant difference in early recurrence between the two groups (106/215 vs. 124/215, *P* = 0.082), while the late recurrence rate in the AVT group was significantly lower than that in the non-AVT group (39/109 vs. 61/91, *P* = 0.012).

## Discussion

In this study, viral reactivation occurred in 10.8% (58/538) of patients who underwent PRFA for HBV-related HCC**.** We demonstrated that AVT could inhibit viral reactivation, decrease tumor recurrence, especially late relapse after 2 years of PRFA and recurrence in the distant hepatic segment, and improve the overall survival.

In HBV-infected patients, viral reactivation frequently occurred spontaneously or more commonly triggered by immune suppression due to various reasons [19]. In these patients with malignancies, the rate of viral reactivation is increased following some anti-cancer treatments, which has been observed in patients with lymphoma who were treated with rituximab and in patients with breast cancer who received post-resection anthracycline-based adjuvant chemotherapy [20, 21]. Reactivation was also observed after kidney and liver transplantation [22, 23]. More observations were conducted in HBV-related HCC patients, showing that viral reactivation was common after partial hepatectomy, even for patients with a low preoperative HBV-DNA level of < 2000 IU/mL, and also after TACE [21, 24]. Dan et al. reported only 5.6% of patients (7/125) with HBV reactivation after RFA which was about half of our finding. The difference may attribute to the variety in time span for observing viral reactivation (2 weeks in Dan’s study and 3 months in our study, respectively). The possible reason for viral reactivation after PRFA remains to be determined. A systemic inflammatory response could be caused by ablation, which was similar to that caused by surgery [25]. A report showed that the serum concentration of interleukin-6 (IL-6) and IL-10, which play a role in immune suppression, could also be increased after PRFA [26]. The elevated serum IL-6 and hepatocyte growth factor both exert immunosuppressive effects, in patients with colorectal liver metastases who were treated with PRFA or liver resection [27]. Although PRFA is one of the first-line treatment options for HCC with less complications when compared with resection, most of the patients receiving PRFA have relatively severe cirrhosis, old age, and poor general condition [4]. These patients often have weakened immunity, which may increase the possibility of viral activation.

Viral reactivation might be associated with an increased tumor recurrence after liver resection or transplantation for HBV-related HCC [6]. Our results noted the oncological disadvantage of viral reactivation which significantly increased recurrence and was one of the independent factors for recurrence after PRFA. Our data showed that AVT significantly reduced the risk of viral reactivation. Furthermore, compared with patients in the non-AVT group, patients with AVT had decreased recurrence rates. In addition, the recurrence rate was lowest in the AVT group and lower in the non-AVT group with a low viral level than the non-AVT group with a high viral level. AVT was also the only protective factor to decrease recurrence after PRFA regardless of the viral level before PRFA. Accordingly, after PRFA, AVT could reduce viral reactivation and improve prognosis.

There are three recognized mechanisms of recurrence after PRFA for HCC: residual tumor, intrahepatic metastasis (IM), and multicentric origins (MO). Residual tumor results from incomplete ablation and is close to the primary nodule. IM is closely associated with intrahepatic micrometastasis from primary HCC and distributes mainly in adjacent or multiple hepatic segments. MO commonly results in a secondary carcinogenesis (de novo tumor) and locates mostly in the distal hepatic segment. IM or MO mechanism may also contribute to early or late recurrence, respectively. In our study, non-AVT patients suffered from more recurrences in the distant segment than AVT patients. In addition, we also found that the late recurrent rate was significantly lower in patients with AVT, but there was no difference was observed in the early recurrence rate. We thus speculated that the AVT mainly reduced the recurrence through improving the liver microenvironment and reducing de novo tumors.

There are two previous studies concerning the relationship between AVT and recurrence after PRFA [13, 14]. In Lee’s study, the difference in the 3-year recurrence rate between the AVT group and no treatment group was not observed, although the difference in 2-year recurrence was significant, which was mainly due to incomplete ablation. Moreover, the detailed information on HBV viral level and HBeAg status, which are important known factors for the prognosis of HBV-related HCC, was missing [13, 14]. In the other study from Sohn, only patients with a solitary HCC were analyzed and the results could not apply to patients who undergo RFA for multiple HCCs, which was an important factor associated with recurrence [14]. Additionally, the initiation of AVT in Sohn’s study was indicated only when HBV DNA ≥ 2000 IU/mL. However, we recommended AVT for all patients with HBV-related HCC regardless of viral level, according to the guideline of AVT in China. Furthermore, neither of these two studies addressed the impact of viral reactivation and AVT on long-term prognosis after PRFA. As we all know, early-stage HCC patients who were offered with PRFA usually have a long duration of survival, and post-PRFA viral change profile and whether AVT could extend the time to the first recurrence and its effect on the recurrence pattern remain unclear. Our results showed that HBV reactivation after PRFA was observed and the activation increased the risk of tumor recurrence. AVT could reduce the risk by decreasing viral reactivation and inhibit late recurrence and relapse in the distant hepatic segment, highlighting the importance of AVT in the management of HBV-related HCC, even with a minimal invasive method such as PRFA.

Our study has several limitations. Firstly, this is a single-center retrospective study. To minimize potential bias inherent in a non-randomized study, we adopted a PSM analysis to determine the prognostic benefit in patients with AVT; after matching, similar demographic and clinical characteristics were identified in both AVT and non-AVT groups. Second, the information was limited to analyze the correlation between different AVT drugs and long-term prognosis because of its retrospective nature. Further studies with multicentric data or in prospective approach are needed to clarify this issue.

## Conclusions

In conclusion, the present study demonstrated HBV reactivation occurred after PRFA. Viral reactivation was associated with recurrence and OS. AVT could reduce recurrence and improve OS, probably through decreasing the rate of viral reactivation.

## Supplementary Information

**Additional file 1:** **Supplementary Table 1.** AVT medication with nucleos(t)ide analogues. **Supplementary Table 2.** Baseline characteristics of AVT and Non-AVT. **Supplementary Table 3.** Analysis of independent risk factors for AVT. **Supplementary Table 4.** Analysis of independent risk factors for local recurrence. **Supplementary Table 5.** Analysis of independent risk factors for distant recurrence.

## Data Availability

The data that support the findings of this study are available from the corresponding authors upon reasonable request.

## Abbreviations

HCC: Hepatocellular carcinoma
HBV: Hepatitis B virus
PRFA: Percutaneous radiofrequency ablation
AVT: Antiviral therapy
HBsAg: Hepatitis B surface antigen
HBcAb: Hepatitis B core antibody
BCLC: Barcelona Clinic Liver Cancer
AFP: Alpha-fetoprotein
TACE: Transarterial chomoembolization
HCV: Hepatitis C virus
HBV DNA: HBV deoxyribonucleic acid
CT: Computerized tomography
MRI: Magnetic resonance imaging
ECOG: Eastern Cooperative Oncology Group
ALT: Alanine aminotransferase
OS: Overall survival
PSM: Propensity score matching
HBeAg: Hepatitis B e antigen
OR: Odds ratio
HR: Hazard ratio
CI: Confidence interval
IM: Intrahepatic metastasis
MO: Multicentric origins

## References

[1] Llovet JM, Kelley RK, Villanueva A, Singal AG, Pikarsky E, Roayaie S (2021). Hepatocellular carcinoma Nat Rev Dis Primers.

[2] Mysore KR, Leung DH (2018). Hepatitis B and C. Clin Liver Dis.

[3] Chen CJ, Yang HI, Su J, Jen CL, You SL, Lu SN, REVEAL-HBV Study Group (2006). Risk of hepatocellular carcinoma across a biological gradient of serum hepatitis B virus DNA level. JAMA.

[4] Lencioni R (2010). Loco-regional treatment of hepatocellular carcinoma. Hepatology.

[5] N'Kontchou G, Mahamoudi A, Aout M, Ganne-Carrié N, Grando V, Coderc E (2009). Radiofrequency ablation of hepatocellular carcinoma:long-term results and prognostic factors in 235 Western patients with cirrhosis. Hepatology.

[6] Qu LS, Jin F, Huang XW, Shen XZ (2010). High hepatitis B viral load predicts recurrence of small hepatocellular carcinoma after curative resection. J Gastrointest Surg.

[7] Kusumoto S, Tanaka Y, Mizokami M, Ueda R (2011). Clinical significance of hepatitis B virus (HBV)-DNA monitoring to detect HBV reactivation after systemic chemotherapy. J Clin Oncol.

[8] Chou CH, Chen PJ, Lee PH, Cheng AL, Hsu HC, Cheng JC (2007). Radiation-induced hepatitis B virus reactivation in liver mediated by the bystander effect from irradiated endothelial cells. Clin Cancer Res.

[9] Kubo S, Nishiguchi S, Hamba H, Hirohashi K, Tanaka H, Shuto T (2001). Reactivation of viral replication after liver resection in patients infected with hepatitis B virus. Ann Surg.

[10] Knoll A, Boehm S, Hahn J, Holler E, Jilg W (2004). Reactivation of resolved hepatitis B virus infection after allogeneic haematopoietic stem cell transplantation. Bone Marrow Transplant.

[11] Yoo S, Lee D, Shim JH, Kim KM, Lim YS, Lee HC (2022). Risk of hepatitis B virus reactivation in patients treated with immunotherapy for anti-cancer treatment. Clin Gastroenterol Hepatol.

[12] Lei Z, Xia Y, Si A, Wang K, Li J, Yan Z (2018). Antiviral therapy improves survival in patients with hbv infection and intrahepatic cholangiocarcinoma undergoing liver resection. J Hepatol.

[13] Lee TY, Lin JT, Zeng YS, Chen YJ, Wu MS, Wu CY (2016). Association between nucleos(t)ide analog and tumor recurrence in hepatitis B virus-related hepatocellular carcinoma after radiofrequency ablation. Hepatology.

[14] Sohn W, Kang TW, Choi SK, Jung SH, Lee MW, Lim HK (2016). Effect of oral antiviral treatment on long-term outcomes of radiofrequency ablation therapy for hepatitis B virus-related hepatocellular carcinoma. Oncotarget.

[15] Bruix J, Sherman M (2011). American Association for the Study of Liver Diseases Management of hepatocellular carcinoma: an update. Hepatology.

[16] Peng ZW, Zhang YJ, Chen MS, Xu L, Liang HH, Lin XJ (2013). Radiofrequency ablation with or without transcatheter arterial chemoembolization in the treatment of hepatocellular carcinoma: a prospective randomized trial. J Clin Oncol.

[17] Wang C, Wang H, Yang W, Hu K, Xie H, Hu KQ (2015). Multicenter randomized controlled trial of percutaneous cryoablation versus radiofrequency ablation in hepatocellular carcinoma. Hepatology.

[18] Wang K, Liu J, Yan ZL, Li J, Shi LH, Cong WM (2010). Overexpression of aspartyl-(asparaginyl)-beta-hydroxylase in hepatocellular carcinoma is associated with worse surgical outcome. Hepatology.

[19] Dan JQ, Zhang YJ, Huang JT, Chen MS, Gao HJ, Peng ZW (2013). Hepatitis B virus reactivation after radiofrequency ablation or hepatic resection for HBV-related small hepatocellular carcinoma: a retrospective study. Eur J Surg Oncol.

[20] Huang YH, Hsiao LT, Hong YC, Chiou TJ, Yu YB, Gau JP (2013). Randomized controlled trial of entecavir prophylaxis for rituximab-associated hepatitis B virus reactivation in patients with lymphoma and resolved hepatitis B. J Clin Oncol.

[21] Yun J, Kim KH, Kang ES, Gwak GY, Choi MS, Lee JE (2011). Prophylactic use of lamivudine for hepatitis B exacerbation in post-operative breast cancer patients receiving anthracycline-based adjuvant chemotherapy. Br J Cancer.

[22] Garg H, Sarin SK, Kumar M, Garg V, Sharma BC, Kumar A (2011). Tenofovir improves the outcome in patients with spontaneous reactivation of hepatitis B presenting as acute-on-chronic liver failure. Hepatology.

[23] Hwang JP, Feld JJ, Hammond SP, Wang SH, Alston-Johnson DE, Cryer DR (2020). Hepatitis B virus screening and management for patients with cancer prior to therapy: ASCO provisional clinical opinion update. J Clin Oncol.

[24] Jang JW, Choi JY, Bae SH, Kim CW, Yoon SK, Cho SH (2004). Transarterial chemo-lipiodolization can reactivate hepatitis B virus replication in patients with hepatocellular carcinoma. J Hepatol.

[25] Shi L, Wang J, Ding N, Zhang Y, Zhu Y, Dong S (2019). Inflammation induced by incomplete radiofrequency ablation accelerates tumor progression and hinders PD-1 immunotherapy. Nat Commun.

[26] Erinjeri JP, Thomas CT, Samoilia A, Fleisher M, Gonen M, Sofocleous CT (2013). Image-guided thermal ablation of tumors increases the plasma level of interleukin-6 and interleukin-10. J Vasc Interv Radiol.

[27] Hinz S, Tepel J, Röder C, Kalthoff H, Becker T (2015). Profile of serum factors and disseminated tumor cells before and after radiofrequency ablation compared to resection of colorectal liver metastases - a pilot study. Anticancer Res.

