# Supplementary material for: Antiviral therapy inhibited HBV-reactivation and improved long-term outcomes in patients who underwent radiofrequency ablation for HBV-related hepatocellular carcinoma
Source: World J Surg Oncol. 2023 Feb 11;21:42. doi: 10.1186/s12957-023-02921-1 (PMC9921597; doi:10.1186/s12957-023-02921-1)
Supplement: Supplementary file 1 — Additional file 1: Supplementary Table 1. AVT medication with nucleos(t)ide analogues. Supplementary Table 2. Baseline characteristics of AVT and Non-AVT. Supplementary Table 3. Analysis of independent risk factors for AVT. Supplementary Table 4. Analysis of independent risk factors for local recurrence. Supplementary Table 5. Analysis of independent risk factors for distant recurrence. [file 12957_2023_2921_MOESM1_ESM.docx]

**Supplementary Table 1. AVT medication with nucleos(t)ide analogues**

| **Drugs** | **Pre-PRFA***  **(n=56)** | **Post-PRFA****  **(n=156)** | **Post-viral reactivation*** (n=28)** |
| --- | --- | --- | --- |
| Entecavir | 15 | 75 | 11 |
| Lamivudine | 14 | 31 | 8 |
| Lamivudine + Entecavir | 3 | 11 | 2 |
| Adefovir | 11 | 18 | 3 |
| Lamivudine + Adefovir | 13 | 21 | 4 |

* receiving AVT within 1 year before PRFA.

** receiving AVT immediately after PRFA.

*** receiving AVT after HBV reactivation.

**Supplementary Table 2. Baseline characteristics of AVT and Non-AVT**

| **Variable** | **Median (IQR)/Number** | | ***P*** |
| --- | --- | --- | --- |
|  | **AVT(n=240)** | **Non-AVT (n=298)** |  |
| Age, years | 55 (29-70) | 54 (28-70) | 0.536 |
| Sex, female: male | 36: 204 | 45: 253 | 0.974 |
| Diabetes mellitus, yes: no | 37: 203 | 34: 264 | 0.172 |
| ECOG, 0: 1: 2 | 112: 121: 7 | 127: 164: 7 | 0.552 |
| AFP, μg/L≥20, yes: no | 111: 129 | 135: 163 | 0.826 |
| Total bilirubin, μmol/L | 14.5 (3.5-50.4) | 15.1 (5.7-49.4) | 0.690 |
| Albumin, g/L | 41.3 (27.1-52.8) | 41.8 (24.6-52.1) | 0.545 |
| Platelets, 10^9^/L | 127 (40-372) | 118 (41-295) | 0.477 |
| Prothrombin time, s | 12.5 (10.1-16) | 12.4 (10.3-15.8) | 0.772 |
| GGT, U/L | 49.5 (9-762) | 61 (7-935) | 0.012 |
| ALP, U/L | 82 (16-273) | 85 (29-443) | 0.058 |
| ALT, U/L | 31.1 (7.9-299.0) | 32.5 (6.2-290.1) | 0.966 |
| Creatinine, μmol/L | 66.0 (4.0-155.0) | 68.0 (6.9-291.0) | 0.150 |
| AFU, U/L | 25 (6-57) | 26 (5-60) | 0.411 |
| HBsAg, positive: negative | 235: 5 | 254: 44 | <0.001 |
| HBeAg, positive: negative | 78: 162 | 58: 240 | 0.001 |
| HBV-DNA, IU/mL, ≥2000:<2000 | 98: 142 | 134: 164 | 0.336 |
| Tumor number, single: multiple | 180: 60 | 229: 69 | 0.618 |
| Liver cirrhosis, yes: no | 58: 182 | 68: 230 | 0.714 |
| Diameter, cm, ≤3:>3 | 2.4 (0.8-5.0) | 2.4 (0.9-5.0) | 0.137 |
| BCLC staging, 0: A | 66: 174 | 71: 227 | 0.331 |
| HBV reactivation, yes: no | 31: 209 | 27: 271 | 0.152 |

Abbreviations: IQR, interquartile range; AVT, antiviral therapy; ECOG: Eastern Cooperative Oncology Group score standard; AFP, Alpha-fetoprotein; GGT, **gamma-glutamyl transferase; ALP,** alkaline phosphatase; ALT, alanine aminotransferase; AFU, α-L- fucosidase; HbsAg, Hepatitis B surface antigen; HBeAg, Hepatitis B e antigen; BCLC, Barcelona Clinic Liver Cancer.

Continuous data are expressed as the median (IQR).

**Supplementary Table 3. Analysis of independent risk factors for AVT**

| **Variable** | **OR** | **P** |
| --- | --- | --- |
| Age, years | 1.003 | 0.987 |
| Sex, female: male | 0.992 | 0.974 |
| Diabetes mellitus, yes: no | 1.415 | 0.174 |
| AFP, μg/L≥20, yes: no | 1.039 | 0.826 |
| Tumor number, single: multiple | 1.106 | 0.618 |
| Liver cirrhosis, yes: no | 1.078 | 0.714 |
| Diameter, cm, ≤3: >3 | 0.659 | 0.056 |
| BCLC staging 0:1 | 0.825 | 0.331 |
| ECOG, 0: 1: 2 | 0.889 | 0.111 |
| Total bilirubin, μmol/L | 0.995 | 0.673 |
| Albumin, g/L | 0.996 | 0.814 |
| Platelets, 10^9^/L | 1.001 | 0.581 |
| Prothrombin time, s | 0.997 | 0.964 |
| GGT, U/L | 0.707 | 0.050 |
| ALP, U/L | 0.993 | 0.003 |
| ALT, U/L | 1.001 | 0.794 |
| Creatinine, μmol/L | 0.993 | 0.126 |
| AFU, U/L | 0.995 | 0.577 |
| HBsAg, positive: negative | 8.142 | <0.001 |
| HBeAg, positive: negative | 1.992 | 0.001 |
| HBV-DNA, IU/mL, ≥2000: <2000 | 0.845 | 0.336 |
| HBV reactivation, yes: no | 1.489 | 0.154 |

Abbreviations: AVT, antiviral therapy; OR, odd ratio; AFP, alpha fetoprotein; ECOG: Eastern Cooperative Oncology Group score standard; GGT, **gamma-glutamyl transferase; ALP,** alkaline phosphatase; ALT, alanine aminotransferase; AFU, α-L-fucosidase; HbsAg, Hepatitis B surface antigen; HBeAg, Hepatitis B e antigen.

**Supplementary Table 4. Analysis of independent risk factors for local recurrence**

| **Variables** | **Univariate** | | | **Multivariate** | | |
| --- | --- | --- | --- | --- | --- | --- |
|  | **HR** | **95%CI** | **P** | **HR** | **95%CI** | **P** |
| Age, years | 1.236 | 0.502-3.039 | 0.645 |  |  |  |
| Sex, female: male | 1.108 | 0.143-8.573 | 0.922 |  |  |  |
| Diabetes mellitus, yes: no | 0.353 | 0.099-1.263 | 0.109 |  |  |  |
| AFP, μg/L≥20, yes: no | 1.072 | 0.409-2.807 | 0.888 |  |  |  |
| Tumor number, single: multiple | 0.677 | 0.267-1.715 | 0.411 |  |  |  |
| Liver cirrhosis, yes: no | 0.354 | 0.132-0.952 | 0.040 | 0.483 | 0.176-1.320 | 0.156 |
| Diameter, cm, ≤3: >3 | 2.914 | 1.553-5.471 | 0.001 | 2.664 | 1.425-4.980 | 0.002 |
| ECOG, 0: 1: 2 | 0.636 | 0.237-1.712 | 0.371 |  |  |  |
| Total bilirubin, μmol/L | 0.983 | 0.326-2.970 | 0.976 |  |  |  |
| Albumin, g/L | 0.766 | 0.173-3.392 | 0.726 |  |  |  |
| Platelets, 10^9^/L | 0.742 | 0.282-1.949 | 0.545 |  |  |  |
| Prothrombin time, s | 1.357 | 0.549-3.352 | 0.508 |  |  |  |
| GGT, U/L | 1.732 | 0.652-4.602 | 0.270 |  |  |  |
| ALP, U/L | 1.140 | 0.327-3.980 | 0.837 |  |  |  |
| ALT, U/L | 0.814 | 0.293-2.266 | 0.694 |  |  |  |
| Creatinine, μmol/L | 1.012 | 0.959-1.067 | 0.665 |  |  |  |
| AFU, U/L | 1.062 | 0.984-1.146 | 0.125 |  |  |  |
| HBsAg, positive: negative | NA | NA | NA |  |  |  |
| HBeAg, positive: negative | 0.998 | 0.411-2.425 | 0.997 |  |  |  |
| HBV-DNA, IU/mL, ≥2000: <2000 | 0.579 | 0.238-1.410 | 0.229 |  |  |  |
| AVT, yes: no | 0.691 | 0.280-1.705 | 0.423 |  |  |  |
| HBV reactivation, yes: no | 1.612 | 0.565-4.602 | 0.372 |  |  |  |

Abbreviations: HR, hazard ratio; CI, confidence interval; AFP, alpha fetoprotein; ECOG: Eastern Cooperative Oncology Group score standard; GGT, **gamma-glutamyl transferase; ALP,** alkaline phosphatase; ALT, alanine aminotransferase; AFU, α-L-fucosidase; HbsAg, Hepatitis B surface antigen; HBeAg, Hepatitis B e antigen; AVT, antiviral therapy.

**Supplementary Table 5. Analysis of independent risk factors for distant recurrence**

| **Variables** | **Univariate** | | | **Multivariate** | | |
| --- | --- | --- | --- | --- | --- | --- |
|  | **HR** | **95% CI** | **P** | **HR** | **95% CI** | **P** |
| Age, years | 1.106 | 0.880-1.389 | 0.389 |  |  |  |
| Sex, female: male | 1.120 | 0.813-1.543 | 0.490 |  |  |  |
| Diabetes mellitus, yes: no | 1.207 | 0.876-1.663 | 0.250 |  |  |  |
| AFP, μg/L≥20, yes: no | 1.039 | 0.826-1.308 | 0.741 |  |  |  |
| Tumor number, single: multiple | 1.563 | 1.208-2.023 | 0.001 | 1.467 | 1.129-1.906 | 0.004 |
| Liver cirrhosis, yes: no | 1.805 | 1.403-2.323 | <0.001 | 1.757 | 1.362-2.267 | <0.001 |
| Diameter, cm, ≤3: >3 | 1.037 | 0.906-1.187 | 0.599 |  |  |  |
| BCLC staging 0: 1 | 0.991 | 0.767-1.281 | 0.944 |  |  |  |
| ECOG, 0: 1: 2 | 1.047 | 0.850-1.290 | 0.665 |  |  |  |
| Total bilirubin, μmol/L | 1.036 | 0.801-1.339 | 0.787 |  |  |  |
| Albumin, g/L | 0.978 | 0.955-1.002 | 0.064 |  |  |  |
| Platelets, 10^9^/L | 0.998 | 0.995-1.002 | 0.057 |  |  |  |
| Prothrombin time, s | 1.150 | 0.899-1.471 | 0.267 |  |  |  |
| GGT, U/L | 1.129 | 0.897-1.421 | 0.302 |  |  |  |
| ALP, U/L | 1.090 | 0.759-1.566 | 0.640 |  |  |  |
| ALT, U/L | 1.030 | 0.806-1.316 | 0.812 |  |  |  |
| Creatinine, μmol/L | 0.808 | 0.334-1.956 | 0.637 |  |  |  |
| AFU, U/L | 1.048 | 0.658-1.669 | 0.843 |  |  |  |
| HBsAg, positive: negative | 5.822 | 1.448-23.405 | 0.013 | 4.170 | 1.030-16.881 | 0.045 |
| HBeAg, positive: negative | 0.988 | 0.759-1.286 | 0.930 |  |  |  |
| HBV-DNA, IU/mL, ≥2000: <2000 | 1.555 | 1.235-1.958 | <0.001 | 1.408 | 1.107-1.791 | 0.005 |
| AVT, yes: no | 0.605 | 0.480-0.762 | <0.001 | 0.569 | 0.450-0.719 | <0.001 |
| HBV reactivation, yes: no | 1.609 | 1.147-2.257 | 0.006 | 1.548 | 1.087-2.204 | 0.015 |

Abbreviations: HR, hazard ratio; CI, confidence interval; AFP, alpha fetoprotein; ECOG: Eastern Cooperative Oncology Group score standard; GGT, gamma-glutamyl transferase; ALP, alkaline phosphatase; ALT, alanine aminotransferase; AFU, α-L-fucosidase; HbsAg, Hepatitis B surface antigen; HBeAg, Hepatitis B e antigen; AVT, antiviral therapy
